# Art in Medicine Curriculum: Improving Empathy and Wellness in Pediatric Hematology-Oncology Fellows

**DOI:** 10.7759/cureus.94127

**Published:** 2025-10-08

**Authors:** Rachel Gallant, Cassandra Wang, Jamie Stokke

**Affiliations:** 1 Pediatric Hematology-Oncology, University of Oklahoma Health Sciences Center, Oklahoma City, USA; 2 Cancer and Blood Disease Institute, Children's Hospital Los Angeles, Los Angeles, USA

**Keywords:** art curriculum, art in medicine, medical education, trainee empathy, trainee well-being

## Abstract

Background: Art curricula have been introduced into medical education to improve observational skills, allow for reflection, and improve empathy and creativity at the medical school, residency, and faculty levels. We aimed to improve the wellness, empathy, and appreciation of art's intersection with medicine of pediatric hematology-oncology fellows through an art in medicine curriculum.

Methods: In 2021-2022, an art curriculum was developed according to the interests of the participating trainees as measured on a needs assessment. Surveys administered prior to implementation and at the end of the 12-month period assessed fellows' views of the role of art in medicine, current usage of art to improve wellness, and how art exposure can impact practitioners' delivery of care, wellness, and empathy.

Results: Twelve fellows were eligible and all participated in the curriculum; 12/12 (100%) completed the pre-intervention survey, and 7/12 (58%) completed the post-intervention survey. Most fellows (57%) reported that they were able to incorporate art into their lives more routinely after participating in the art curriculum, and 86% reported a change in their clinical practice. Fellows reported more empathy and creativity in clinical practice and strengthened bonds with colleagues. Fellow satisfaction with program initiatives to promote wellness also improved from 58% to 85%.

Conclusion: Implementation of a longitudinal art curriculum is feasible and demonstrates benefit to pediatric hematology-oncology fellows.

## Introduction

Well-being of medical trainees is crucial to developing effective, well-rounded physicians and reducing burnout. Burnout affects physicians at all levels of training and across all specialties. There are multiple theories of how burnout develops, but all include similar themes of demands outweighing rewards in both the technical and emotional aspects of the job. Pediatric hematology-oncology (PHO) can be especially emotionally draining, and therefore, PHO physicians are at risk for burnout. In a review by Whitford et al., up to 72% of PHO physicians reported symptoms of burnout with nearly 40% experiencing high levels of burnout [1]. Furthermore, risk factors included having fewer than 10 years of experience [1] raising concern that PHO trainees may be at higher risk as they are early in their career. Moerdler et al. surveyed PHO fellows from over 20 training programs and found 39% were experiencing high levels of burnout [2]. Not only is this troublesome for the well-being of PHO physicians, but also there are well-established adverse effects of burnout that impact patients and the greater healthcare system. Burnout increases the rate of medical errors, affects the physician-patient relationship and patient satisfaction, and can lead to increased turnover of personnel and sick leave, ultimately increasing financial burden for health systems [1]. As such, it is crucial that we place an emphasis on PHO physicians' well-being starting at the trainee level.

Ensuring trainee well-being is now a program requirement of the Accreditation Council for Graduate Medical Education (ACGME) [3]. On our institutional PHO program survey, only 58% of trainees were satisfied with the program's effort to promote trainee well-being, and we scored below the national average on the ACGME well-being questionnaire. Given the importance of trainee well-being and the unique challenges of PHO, our fellows and faculty recognize the importance of improving our program's trainee wellness initiatives.

Art curricula have been introduced into medical education at the medical school, residency, and faculty levels and typically involve visual arts-based training which has been shown to improve the observational skills of medical students and residents [4,5]. Art curricula also create an opportunity for reflection [6-8], may prevent burnout, and improve trainee empathy and creativity [7,9,10]. Most art curricula in medical education are largely visual arts-based, are implemented as short-duration curricula, and occur in undergraduate medical education. To further evaluate the effect of art in medicine on medical trainees, we launched a pilot study implementing a longitudinal art curriculum for PHO fellows tailored to trainees' interests with the goal of improving trainee wellness.

## Materials and methods

Twelve PHO fellows at Children's Hospital Los Angeles (CHLA), Los Angeles, CA, USA, participated in the curriculum with approval from the CHLA Institutional Review Board from 2021 to 2022 (approval number: CHLA-21-00385). The cohort included first-, second-, and third-year fellows. Time in fellowship ranged from eight to 32 months at the time of curriculum implementation. The learner level was considered beginner to advanced beginner as most of the fellows had not participated in an art in medicine curriculum prior to this study [11]. The cohort was ethnically diverse including African-American, Asian/Pacific Islander, and non-Hispanic White participants; two international medical graduates; and two who were first generation born in the United States.

An art curriculum was developed following Kern's six-step approach to curriculum development [12]. Activities were trainee-planned including the art forms that were most meaningful to the trainees as described in the needs assessment. Trainees led each session for a total of 10 sessions, approximately one per month, which encompassed various art forms. Activities included the following: (i) collaborative playlist creation where trainees were asked to share meaningful songs with one another, (ii) collaborative photo collage in which trainees took photos of things in their everyday life and compiled them into a collage sharing the meaning of each of their photos, (iii) instructor-led painting where an outside painting instructor led fellows in a painting workshop, (iv) collaborative cookbook creation where fellows, faculty, and staff contributed favorite recipes and shared what meaning that recipe holds for them, (v) virtual cooking classes led by our program director allowing fellows to cook at home with their friends or family, (vi) pumpkin painting, (vii) drawing/reflection exercise where fellows were asked to draw a pie chart representing their most important relationships, and (viii) painting/planting flowerpots. All activities were designed to be completed in 1-2 hours including a structured debrief. Debrief questions included "What did you learn from this activity?"; "Does this resonate as a stress reliever or coping mechanism?"; "Does this shape how you could incorporate art into your medical practice?"; "What did you learn about your colleagues?"; and "How does this change your view of art in medicine?". Activities took place during regularly scheduled lecture periods for fellows. Occasional activities were held during the annual PHO fellow retreat, and virtual cooking classes were offered in the evenings. Activities were offered to all PHO fellows but attendance was not required.

This was a mixed-methods study utilizing non-paired pre- and post-intervention survey data and qualitative free-response questions (see Appendix A). Anonymous pre-intervention surveys were designed to identify meaningful art forms and assess fellows' views of the role of art in medicine and how art exposure can impact practitioners' delivery of care. At the end of the 12-month period, follow-up surveys assessed attitudes regarding the curriculum and behavior change by asking about changes in fellows' views of art in medicine and/or their clinical practice. All surveys were administered electronically, and multiple e-mail reminders were sent to encourage survey completion.

Free-response data were categorized using inductive thematic analysis following Braun and Clarke's six-step approach [13,14]. Two reviewers (RG, JS) evaluated the qualitative data to determine and agree on themes. The chi-squared test was performed to compare categorical data before and after the implementation of the art curriculum using the Stata 16 software (StataCorp LLC, College Station, TX, USA) [15].

## Results

**Figure 1:**
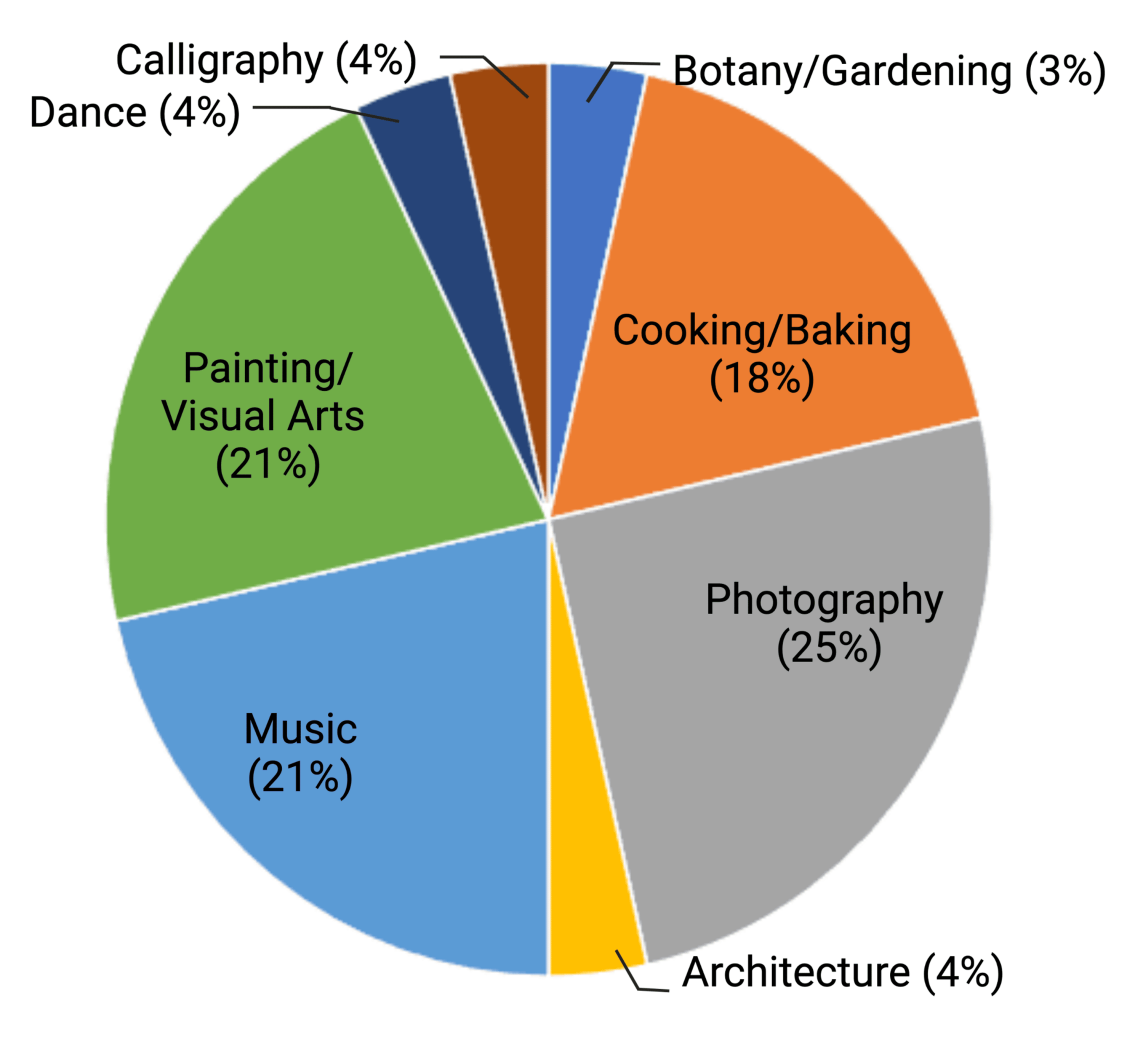
Meaningful art forms identified by pediatric hematology-oncology fellows on needs assessment Responses of pediatric hematology-oncology fellows when asked what art forms they find meaningful. Created with BioRender

Twelve fellows were eligible to participate; all fellows (100%) completed the pre-intervention survey and participated in the curriculum. Typically, 8-12 fellows were in attendance at each session. Seven fellows (58%) completed the post-intervention survey. A needs assessment revealed that nearly all fellows (92%) find art meaningful and enjoy art hobbies but cannot find time to incorporate art into their lives routinely (75%). The needs assessment also identified art forms meaningful to fellows including visual arts, music, photography, and cooking/baking (Figure 1). On the pre-intervention survey, 92% reported that art has a place in medicine, and 75% felt that art impacts the provision of care to patients. Fellows also reported that art promotes provider creativity and empathy, improves healing and wellness in patients and providers, and builds a stronger work environment (Table 1).

**Table 1:** Themes identified from fellow survey responses on the role of art in medicine Results of pre- and post-intervention surveys administered to fellows. Survey questions were free-response, results were categorized into themes, and chi-squared test was performed to compare pre- and post-intervention responses. Bolded items indicate a p-value of <0.05.

	Pre-intervention n (%)	Post-intervention n (%)	OR (95% CI)	P-value
Art promotes empathy	1 (8%)	5 (71%)	27.5 (1.4, 1413.1)	0.004
Art promotes healing and wellness for patients	4 (33%)	NA	NA	NA
Art promotes creativity	2 (17%)	3 (43%)	3.8 (0.3, 57.4)	0.211
Art is a coping mechanism and source of healing/wellness for providers	3 (25%)	4 (57%)	4.0 (0.4, 44,5)	0.161
Art and medicine share philosophical ideology	3 (25%)	NA	NA	NA
Art strengthens bonds with colleagues	1 (8%)	3 (43%)	8.3 (0.4, 471.3)	0.075

On the follow-up survey (n=7), all fellows reported that they enjoyed the art curriculum and found the activities meaningful. Highest-rated activities included the collaborative playlist creation and instructor-led painting session. Over half of fellows (57%) reported a new ability to appreciate art on a smaller scale, identify new art forms, and incorporate art into their lives routinely. Fellow satisfaction with program initiatives to promote wellness improved from 58% to 78% after implementing the curriculum.

Furthermore, 86% of fellows reported a change in their clinical practice including a greater sense of empathy, increased creativity in patient care, a stronger bond with their colleagues, and utilization of art as a coping mechanism in times of stress (Table 1). Participants were asked to describe how the curriculum changed their practice in a free-text response. They reported a deepened sense of empathy as the curriculum provided them with "better tools to relate to patients and families", "helped [them] connect with [their] patients", and improved their ability "to communicate about music and art that is helpful for coping with difficult situations". Fellows also reported that the curriculum "helped [them] express a more creative side of [themselves]" making them "more creative in [their] decision-making for patient care". The curriculum provided "the space to bond with [their] co-fellows in a different way" and an opportunity to "know [their] colleagues better" and "learn more about [their] coworkers… and how they cope with difficult patient encounters". On the post-intervention survey, fellows were also asked to reflect on a specific difficult patient encounter and whether they might have coped differently after participating in the art curriculum. Responses included the following: "I think that I now have a better understanding of how I processed a patient death in the past. I now am more thoughtful about what music offers me in difficult times or times of stress and can utilize it more fully and therapeutically" and "I found that I actively sought out and scheduled an opportunity to paint in a formal setting after a difficult patient situation. That ended up being incredibly therapeutic and gave me some time and space to process what had happened. I don't think I would have made quite as much of an effort to utilize an art form to process my emotions without this art curriculum reminding me how important and helpful it is to have art as a medium to process difficult experiences".

## Discussion

Our longitudinal art curriculum demonstrated benefit to PHO fellows. Fellows found the art activities enjoyable and reported that art in medicine impacted their clinical practice. Fellows reported a new recognition of certain activities as art forms that they previously did not consider to be "art" such as cooking, baking, and gardening. They also expressed an ability to appreciate art on a smaller scale on a routine basis.

Our art curriculum is unique in that art activities included but were not limited to fellows' specific interests within the arts. Recognizing that not all art forms are meaningful to all individuals, the curriculum was designed to include a variety of art forms in hopes of giving each fellow the opportunity to participate in an activity relevant to their interests while still gaining exposure to other art forms that they may not have experienced before. Unlike many other art in medicine curricula [4,5,7], ours provided ongoing exposure to art over the course of a year.

We found the curriculum feasible to implement in terms of time and cost. Most art activities took place over the noon hour on weekdays, a time typically reserved for fellow educational sessions, while a few activities occurred outside the workday. Fellows were encouraged to bring their lunch to the sessions as is customary at our institution for noon lectures. Most activities require minimal basic art supplies (or no supplies). The instructor-led painting was the most expensive activity, particularly for a large group, but given the variety of activities available, the curriculum can be tailored to fit nearly any budget.

Participation was optional for all activities to avoid undue stress by requiring fellows doing research off-campus to return for these activities. We noted that though participation was voluntary, most fellows participated in each activity except in the event of scheduling conflicts. Even so, only seven fellows completed the post-intervention survey. Surveys were delivered electronically to allow the flexibility for fellows to complete them on their own time from a phone or computer, and they received multiple reminders via e-mail to complete the surveys. We suspect that the suboptimal completion rate of the post-intervention survey may be due to e-mail and survey fatigue as trainees are required to complete many surveys outside of this curriculum. Furthermore, surveys were anonymous, and given the voluntary nature of this study, completion of the surveys could not be mandated. However, for future replication studies, it may be beneficial to administer surveys on paper during designated periods for in-person fellow education to help ensure optimal survey completion.

Though wellness was not objectively measured, qualitative evidence suggests that participation in the curriculum improved fellow wellness. Satisfaction scores on program efforts to promote wellness improved on the ACGME survey after a few months. Though this could be due solely to fellows' perception that the program was taking an interest in their well-being (rather than a result of the curriculum itself), we believe the curriculum played a role in cultivating wellness in our program. Specifically, fellows reported strengthened bonds with co-fellows/colleagues by experiencing art together affording fellows an enhanced support system. Furthermore, some fellows identified art as a new coping mechanism in times of stress (work-related or personal) and felt more emotionally available for their loved ones.

Importantly, fellows noted clinical practice changes. Fellows identified art as a mechanism to relate to patients and their families as well as colleagues. They saw a new role of art in medicine extending beyond typical art therapy, noting that art exposure "brings out the humanistic side of medicine" making more well-rounded providers. Furthermore, they noted a new sense of empathy for their patients and families and felt that they could tackle difficult patient situations with more creativity. 

Though these results are encouraging, we acknowledge our pilot study's limitations. This is a small sample including only PHO fellows, and survey questionnaires were not designed to objectively measure fellow wellness. Furthermore, the response rate on the follow-up survey was less than desired despite multiple e-mail reminders to complete surveys. This may be due to e-mail fatigue in addition to competing demands for fellows' time. We also acknowledge that given the voluntary nature of participation and survey response, it is possible that those who perceived more benefit from the curriculum could have preferentially responded, thereby introducing bias into the results. 

## Conclusions

These results are exciting and suggestive of art in medicine curricula as a creative and feasible way to improve trainee wellness and cultivate empathy. For future expansions of this art curriculum, we will include a wider variety of art activities and include trainees from other subspecialties within pediatrics as well as other levels of trainees. Objective measurements of trainee well-being will also be implemented in future iterations of the curriculum. Given the difficulty with follow-up survey completion in this pilot study, we plan to provide incentives for survey completion to improve response rates. Furthermore, incentivizing survey responses may allow for longer follow-up to evaluate whether trainees have a sustained interest in the arts, improved well-being, and empathy months after the completion of the curriculum. We hope that future studies will provide further evidence of the benefits of a feasible and generalizable art in medicine curriculum for medical trainees. 

## Appendices

Appendix A

Pre-curriculum Survey

Is there a particular type of art (visual art, photography, music, poetry, cooking, etc.) in which you find meaning? If so, please share the types of art that bring you joy.

If art is meaningful to you, are you able to find ways to appreciate or incorporate art in your life on a routine or daily basis? If so, please elaborate.

Do you believe that art has a place (or could have a place) in medicine? If so, please feel free to share any ideas about why or how.

Do you believe that exposure to art and artistic expression can impact how medical providers practice medicine and deliver care to patients?

Have you had any experience with incorporating art in medicine or medical curriculum? If so, please elaborate.

Follow-Up Survey

Did you find the activities in the art in medicine curriculum enjoyable?

Do you feel that you have gained something from participating in the art in medicine sessions? If so, what do you feel you have gained?

Is there a particular session that you found most meaningful? If so, why was it meaningful?

Do you feel that as a result of participating in the art in medicine curriculum you are able to find ways to appreciate art or incorporate art into your life on a routine or daily basis?

Do you believe that art has a place in medicine? If so, has the curriculum changed or strengthened how you view the role of art in medicine?

On a scale of 1-5 (1: unchanged; 2: slightly changed; 3: some change; 4: moderately changed; 5: significantly changed), how much do you feel that your practice of medicine has changed as a result of this curriculum?

If your medical practice has changed, describe in what ways it has changed (e.g., empathy, relating to patients/families, observational skills, creativity).

Think back to a particular difficult encounter with a patient (e.g., a patient's death) and how you processed/reacted to that experience. After participating in the art curriculum, do you feel that you would have reacted or processed that experience differently?

Select the activities that you would like to continue in future sessions (select all that apply): instructor-led painting session, collaborative playlist, pumpkin painting, five most important relationships (pie chart activity), virtual cooking class, and photography collage.

Debrief Questions

What did you learn from this activity?

Does this resonate as a stress reliever or coping mechanism?

Does this shape how you could incorporate art into your medical practice?

What did you learn about your colleagues?

How does this change your view of art in medicine?
